# Two new species of *Paramesosciophilodes* (Diptera, Nematocera, Mesosciophilidae) from the Middle Jurassic of China

**DOI:** 10.3897/zookeys.511.8425

**Published:** 2015-07-02

**Authors:** Jiaqi Gao, Guifeng Shi, Chungkun Shih, Dong Ren

**Affiliations:** 1 College of Life Sciences, 105 Xisanhuanbeilu, Haidian District, Capital Normal University, Beijing 100048, China

**Keywords:** *Paramesosciophilodes*, Daohugou, Inner Mongolia, China

## Abstract

Two new species, *Paramesosciophilodes bellus* **sp. n.** and *Paramesosciophilodes rarissima* **sp. n.**, from the Jiulongshan Formation at Daohugou Village, Inner Mongolia, China, are described in the extinct family Mesosciophilidae. Altogether seven genera with 21 species of mesosciophilids have been described from the Jurassic of Siberia and Kazakhstan, the Lower Cretaceous of Transbaikalia, and the Middle Jurassic of Inner Mongolia. An emended generic diagnosis of *Paramesosciophilodes* and a list of known taxa of mesosciophilids are provided.

## Introduction

Mesosciophilidae is one of the extinct dipteran families of the suborder Nematocera. Rohdendorf (1946) described a species, *Mesosciophila venosa*, which was assigned to a new subfamily, Mesosciophilinae, within the family Allactoneuridae, along with Fungivoritinae. Later he implicitly synonymized Mesosciophilinae with Fungivoritidae and excluded *Allactoneura* DeMejere, 1907 from the family (Rohdendorf 1957, 1962). Kovalev (1985) elevated Mesosciophilinae to family level; and synonymized Fungivoritidae under the First Reviser Rule. Blagoderov (1993) erected the genus *Mesosciophilopsis* with three species within the family Mesosciophilidae, and also revised the diagnosis of the Mesosciophilidae. Two important generic characters of *Mesosciophilina* Kovalev, 1985, reported from the Middle Jurassic, are cell r distinctly large, longer than 1/6 of wing length, and r-m significantly shorter than bRs, which are regarded as “obvious ancestral characters” (Kovalev 1985). On the other hand, the generic features of *Mesosciophilopsis* Blagoderov, 1993, described from the Early Cretaceous, are cell r distinctly small, shorter than 1/6 of wing length, and r-m significantly longer than bRs, which are regarded as “derived characters” (Blagoderov 1993, Zhang 2002). Zhang (2007) established a monotypic genus *Paramesosciophilodes* for his new species, *Paramesosciophilodes ningchengensis*, and described another species within the genus *Mesosciophila*. The generic diagnosis of *Paramesosciophilodes* includes cell r 0.16–0.18 times as long as wing length, bRs markedly shorter than r-m and R_4+5 _is strongly arched near its midlength. Later, Zhang (2008) assigned three new species to three genera, including *Paramesosciophilodes eximia* Zhang, 2008, and reviewed all the records of mesosciophilids. Li and Ren (2009) described two species of *Jurasciophila* from the late Middle Jurassic Jiulongshan Formation of Daohugou in southeastern Inner Mongolia, China. Species of *Jurasciophila* Li & Ren, 2009 have cell r small, shorter than 1/6 of wing length, and r-m significantly shorter than bRs, which are regarded as “transitional characters” (Li and Ren 2009). Wang et al., in 2012, assigned two species respectively to *Mesosciophila* and *Paramesosciophilodes* of Mesosciophilidae (Wang et al. 2012). Shi et al. recently described a new genus with two new species, *Similsciophila singularis* and *Similsciophila sinuate*, from the late Middle Jurassic of Jiulongshan Formation (Shi et al. 2014). To date, 7 genera and 19 species of mesosciophilids have been described from the Jurassic of Siberia and Kazakhstan, the Lower Cretaceous of Transbaikalia, and the Middle Jurassic of Inner Mongolia, which are summarized in Table 1. In addition, an emended generic diagnosis of *Paramesosciophilodes*, based on the new findings, is provided.

**Table 1.** A list of the described fossil Mesosciophilidae.

Genus	Species	Locality	Age
*Mesosciophila*	*Mesosciophila venosa* Rohdendorf, 1946	Karatau, Chimkent Oblast, Kazakhstan	Karabastau Fm., J_3_
	*Mesosciophila eucalla* Zhang, 2007	Daohugou, Ningcheng, Inner Mongolia, China	Jiulongshan Fm., J_2_
	*Mesosciophila abstracta* Zhang, 2008	Daohugou, Ningcheng, Inner Mongolia, China	Jiulongshan Fm., J_2_
	*Mesosciophila sigmoidea* Wang, Zhao & Ren, 2012	Daohugou, Ningcheng, Inner Mongolia, China	Jiulongshan Fm., J_2_
*Mesosciophilodes*	*Mesosciophilodes augustipennis* Rohdendorf, 1946	Karatau, Chimkent Oblast, Kazakhstan	Karabastau Fm., J_3_
	*Mesosciophilodes similis* Rohdendorf, 1964	Karatau, Chimkent Oblast, Kazakhstan	Karabastau Fm., J_3_
	*Mesosciophilodes synchrona* Zhang, 2008	Daohugou, Ningcheng, Inner Mongolia, China	Jiulongshan Fm., J_2_
*Mesosciophilina*	*Mesosciophilina bolshakovi* Kovalev, 1985	Siberia, Russia	Itat Fm., J_2_
	*Mesosciophilina irinae* Kovalev, 1985	Siberia, Russia	Itat Fm., J_2_
*Mesosciophilopsis*	*Mesosciophilopsis curtus* Blagoderov, 1993	Baissa, Buryat, Yeravnenskiy, Transbaikalia	Zaza Fm., K_1_
	*Mesosciophilopsis expletus* Blagoderov, 1993	Baissa, Buryat, Yeravnenskiy, Transbaikalia	Zaza Fm., K_1_
	*Mesosciophilopsis minor* Blagoderov, 1993	Baissa, Buryat, Yeravnenskiy, Transbaikalia	Zaza Fm., K_1_
*Paramesosciophilodes*	*Paramesosciophilodes ningchengensis* Zhang, 2007	Daohugou, Ningcheng, Inner Mongolia, China	Jiulongshan Fm., J_2_
	*Paramesosciophilodes eximia* Zhang, 2008	Daohugou, Ningcheng, Inner Mongolia, China	Jiulongshan Fm., J_2_
	*Paramesosciophilodes aequus* Wang, Zhao & Ren, 2012	Daohugou, Ningcheng, Inner Mongolia, China	Jiulongshan Fm., J_2_
	*Paramesosciophilodes bellus* Gao, Shi, Shih & Ren, sp. n.	Daohugou, Ningcheng, Inner Mongolia, China	Jiulongshan Fm., J_2_
	*Paramesosciophilodes rarissima* Gao, Shi, Shih & Ren, sp. n.	Daohugou, Ningcheng, Inner Mongolia, China	Jiulongshan Fm., J_2_
*Jurasciophila*	*Jurasciophila curvula* Li & Ren, 2009	Daohugou, Ningcheng, Inner Mongolia, China	Jiulongshan Fm., J_2_
	*Jurasciophila lepida* Li & Ren, 2009	Daohugou, Ningcheng, Inner Mongolia, China	Jiulongshan Fm., J_2_
*Similsciophila*	*Similsciophila singularis* Shi, Shih & Ren, 2014	Daohugou, Ningcheng, Inner Mongolia, China	Jiulongshan Fm., J_2_
	*Similsciophila sinuate* Shi, Shih & Ren, 2014	Daohugou, Ningcheng, Inner Mongolia, China	Jiulongshan Fm., J_2_

(Notes: J_2_-Middle Jurassic, J_3_-Late Jurassic, K_1_-Early Cretaceous)

There have been many transfers and corrections regarding species belonging to the Mesosciophilidae. *Eoboletina gracilis* Rohdendorf, 1946 from the Upper Jurassic of Kazakhstan might belong to the family Mesosciophilidae (Blagoderov 1993). The Mongolian genus *Mesosciophilites* Kovalev, 1985 of the Lower Cretaceous should be transferred to the Mycetophilidae (Blagoderov 1993). The Australian species *Pseudalysiinia fragmenta* Jell & Duncan, 1986 should be transferred to an unnamed genus of Mesosciophilidae rather than to the extant genus of *Pseudalysiinia* Tonnoir, 1929 of Mycetophilidae (Blagoderov 1993), and we agree with this change. The genus *Sciophilites* Kovalev, 1990 from the Lower Cretaceous of Transbaikalia might belong to either the Mesosciophilidae or to the Mycetophilidae (Blagoderov 1993). *Sinosciophila meileyingziensis* Hong, 1992 from the Lower Cretaceous of Kezuo has been transferred to the Sciophilidae (Zhang 2008), but it might be a representative of Mesosciophilidae. The other three species *Liaoxifungivora simplicis* Hong, 1992, *Atalosciophila yanensis* Ren, Lu, Guo & Ji, 1995 and *Huaxiasciophilites jingxiensis* Zhang, Hong & Li, 2001 from the Lower Cretaceous of China might belong to the family Mycetophilidae, rather than to its previous assignment to the family of Pleciofungivoridae or the family Mesosciophilidae (Zhang 2007).

Here, based on a combination of unique wing venational characters of two recently collected specimens, we describe *Paramesosciophilodes bellus* sp. n. and *Paramesosciophilodes rarissima* sp. n. These specimens with bodies and complete wings were collected from the late Middle Jurassic Jiulongshan Formation of Daohugou Village in the Ningcheng County, Chifeng City, southeastern Inner Mongolia, China. Many well-preserved fossil insects have been described from this locality recently (Ren et al. 2010, 2012), such as dipterans, neuropterans, orthopterans, heteropterans, etc. (Zhang et al. 2008, 2011; Wang et al. 2010; Gu et al. 2012; Yao et al. 2012).

## Materials and methods

This study is based on two specimens housed in the Key Lab of Insect Evolution & Environmental Changes, Capital Normal University, Beijing, China (Curator: Dong Ren). The specimens were examined under a LEICA MZ12.5 dissecting microscope. The photos of fossils were taken with a Nikon SMZ1000 stereo microscope. Line drawings were prepared with the aid of CorelDraw 12 graphic software. The method of calculating the ratio of cell r length vs. wing length is as follows: the length of cell r is the length along R_1_, while the length of wing is the length from wing base to wing apex. Wing venation nomenclature follows that of Wootton and Ennos (1989) and Shcherbakov et al. (1995): bRs or dRs = section of R_4+5_ basal or distal to r-m, respectively; bM_1+2_ or dM_1+2_ = section of M_1+2_ basal or distal to r-m, respectively.

## Systematic paleontology

### Order Diptera Linnaeus, 1758 Suborder Nematocera Latreille, 1825 Family Mesosciophilidae Rohdendorf, 1946

#### Genus Paramesosciophilodes

Taxon classificationAnimaliaDipteraMesosciophilidae

Zhang, 2007

##### Type species.

*Paramesosciophilodes ningchengensis* Zhang, 2007.

##### Included species.

Type species; *Paramesosciophilodes eximia* Zhang, 2008; *Paramesosciophilodes aequus* Wang, Zhao & Ren, 2012; *Paramesosciophilodes bellus* Gao, Shi, Shih & Ren sp. n., *Paramesosciophilodes rarissima* Gao, Shi, Shih & Ren, sp. n.

##### Emended diagnosis.

Medium (sized mesosciophilid gnats. Body (including legs) covered with long, dense pubescence. Mesonotum convex. Scutellum sharp, clearly projecting. Wing, Sc_1_ elongate, slightly shorter than one-half of wing length (0.43–0.47 times as long as wing length); Sc_2_ situated distinctly basad to Rs origin, arising near midway between h to Sc_1_ ending; bRs shorter than r-m; R_1_ slightly curved; both R_1_ and R_4+5_ divergent terminally; Rs furcated distad or at level of fork of M_1+2_; R_2+3_ oblique and curved; R_4+5_ arched near its midlength; cell r 0.16–0.19 times as long as wing length; stem of M not developed; M_1+2_ furcated slightly distad, or basad, to level of Sc_1_ ending. Tibiae and tarsi with sparse, short setae.

#### Paramesosciophilodes bellus

Taxon classificationAnimaliaDipteraMesosciophilidae

Gao, Shi, Shih & Ren sp. n.

http://zoobank.org/8FEE85B5-4556-40CA-8B60-B8309F5B1504

Figs 1 , 2

##### Etymology.

The specific name is from the Latin of *bellus*, meaning beautiful and delicate, for the well-preserved and beautiful specimen.

##### Material.

Holotype No. CNU-DIP-NN2013631 p/c, part and counterpart. A well-preserved insect with complete body and two wings but poorly preserved halter, without head, in dorsoventral aspect.

##### Locality and horizon.

Daohugou Village, Shantou Township, Ningcheng County, Inner Mongolia, China, Jiulongshan Formation, late Middle Jurassic.

##### Diagnosis.

The Sc_1_ ending proximad of the midlength of cell r; bRs 0.7 times of the length of r-m; R_4+5 _strongly curved; M_1+2_ forking basad of forking of Rs, and distad of the level of Sc_1_ ending; CuA strongly arched, reaching the posterior margin of the wing markedly basad of Rs forking to R_2+3_ and R_4+5_.

##### Description of holotype.

**Figure 1.**
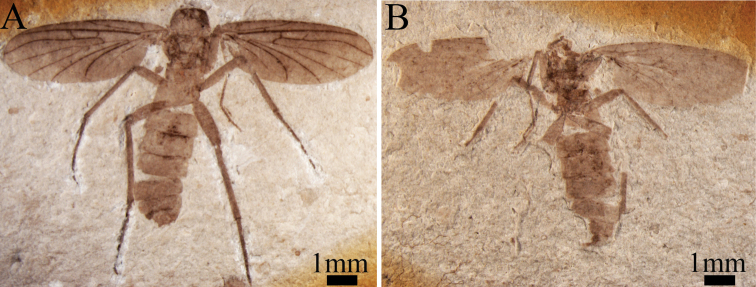
*Paramesosciophilodes bellus* sp. n., holotype, Photographs of habitus (dorsoventral aspect): **A** part No. CNU-DIP-NN2013631 p **B** counterpart CNU-DIP-NN2013631 c.

**Figure 2.**
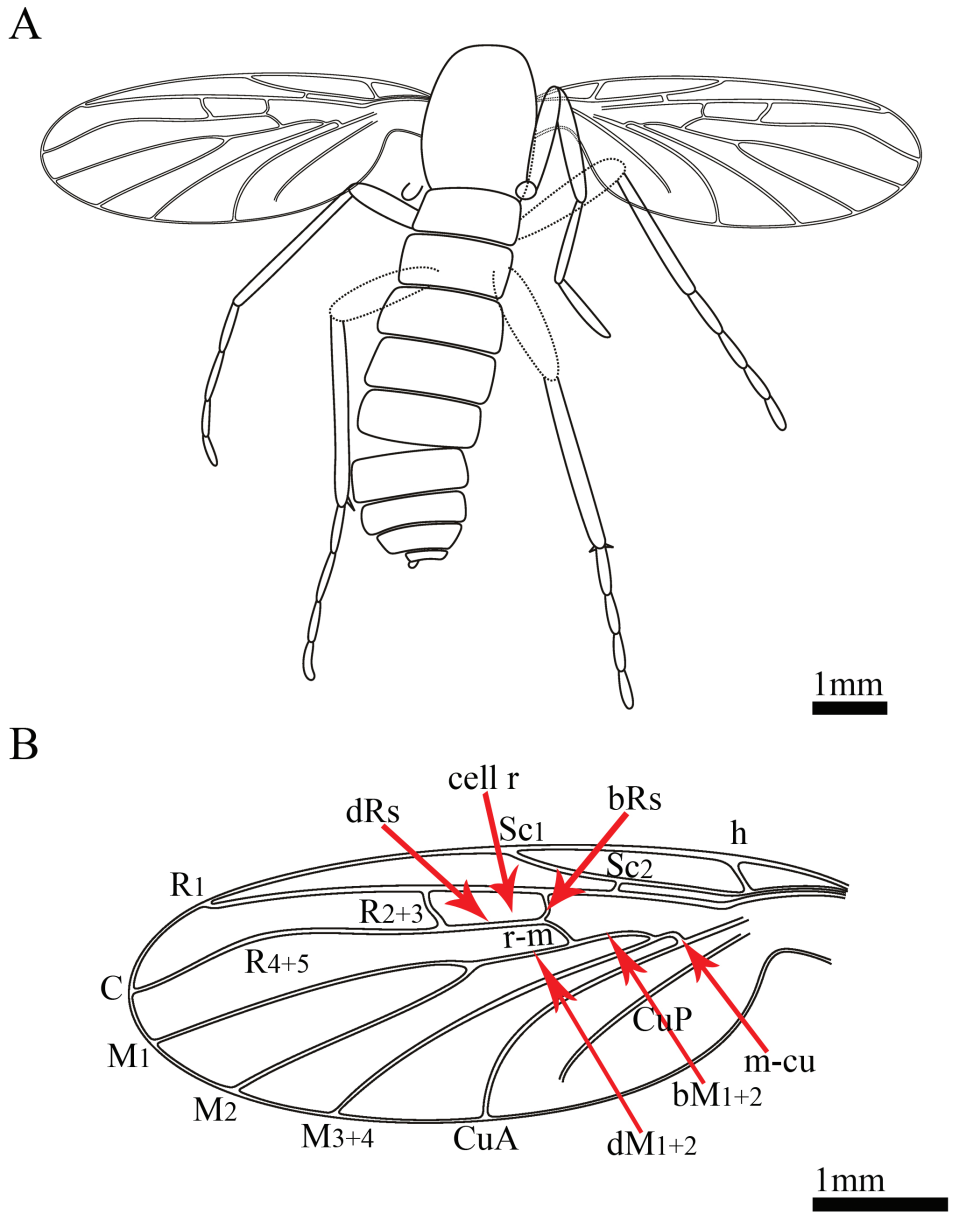
*Paramesosciophilodes bellus* sp. n., Line drawings of holotype: **A** part **B** wing venation.

Medium-sized mesosciophilid with dark body, adult male, in dorsal aspects (Figs 1 and 2A). Wings out-spread, length 5.4 mm, width 2.0 mm. Body length 7.2 mm. Head and antennae not preserved. Thorax convex, length 2.0 mm, width 1.3 mm. Scutellum clearly projecting. Abdomen thin, subcylindrical, length 5.2 mm, width 1.7 mm, approx. 2.6 times as long as head and thorax combined, with eight abdominal segments, first four segments gradually widened distally, last four segments gradually narrowed terminally. Partially preserved male genitalia relatively small, distinctly narrower than eighth abdominal segment. Halters poorly preserved. Legs relatively thin and long, femora clearly thicker in the middle; femora, tibiae and tarsi with two rows of sparse and short setae. Hind leg length 6.3 mm (femur 1.7 mm, tibia 2.4 mm, tarsus 2.2 mm).

Wings membranous, oblong, darker in color in costal area, moderately wide (length 2.7 times of width), and not reaching the apex of abdomen at rest (Fig. 2). C strong, ending beyond wing apex, at which R_4+5_ ending. Sc_1_ relatively long, approx. 0.4 times the length of wing, ending far distad of the intersection of bRs and r-m. Humeral vein distinct and oblique. Sc_2_ well developed, starting in front of bRs. Cell r distinctly small (0.89 mm), approx. 0.165 times the wing length (5.4 mm). Section of R from Sc_2_ to bRs origin approx. 2.4 times as long as bRs. R forking into three branches: R_1_, R_2+3_ and R_4+5_. R_1_ and R_4+5_ somewhat divergent terminally; R_2+3_ and R_4+5_ arched. Forking of Rs distad of the level of M forking. Rs strong, arising from beyond the basal one-third of length of wing, bRs+dRs nearly 0.4 times the R_4+5_. Rs forking to R_2+3_ and R_4+5_ distad of forking of M_1+2_. Section bRs 0.7 times the r-m. R_1_ slightly curved, relatively long (nearly 0.5 times the length of wing), slightly deflected after junction with R_2+3_; R_2+3_ slightly curved, shifted toward wing base, beyond the level of M_1+2_ forking. Vein R_4+5_ strongly arched near its midway, almost parallel with R_1_, but slightly oblique at apex. Stem of M completely reduced basad of crossvein m-cu, with only a short segment distad of m-cu. Stem of M forking into M_1+2_ and M_3+4. _M_1+2_ forking into M_1_ and M_2_ near R_2+3_ level. M_1_ arched anteriorly, M_2_ nearly straight. Crossvein r-m short, curved, slightly oblique, shorter than bRs, nearly perpendicular to M_1+2_, almost parallel to R_2+3_, intersected at M_1+2_, forking to bM_1+2_ and dM_1+2_. bM_1+2_ approx. 6.6 times as long as m-cu. dM_1+2_ approx. as long as bM_1+2_, and longer than r-m. CuA running parallel close to M_3+4_ basally. CuA reaching the posterior margin of wing at approx. the same level of M_1+2_ forking to M_1_ and M_2_. CuP short, slightly curved at its midway, not reaching the posterior margin of wing.

##### Remarks.

*Paramesosciophilodes bellus* sp. n. resembles most closely *Paramesosciophilodes ningchengensis*, but can be distinguished from the latter in having Sc_1_ ending at C proximad of the miglength of cell r (vs. at the miglength of cell r for *Paramesosciophilodes ningchengensis*) and CuA reaching the posterior margin of the wing markedly basad of Rs forking to R_2+3_ and R_4+5_ (vs. slightly basad of Rs forking to R_2+3_ and R_4+5_).

This new species is differentiated from *Paramesosciophilodes ningchengensis*, *Paramesosciophilodes eximia*, *Paramesosciophilodes aequus*, and *Paramesosciophilodes rarissima* sp. n. based on a combination of characters listed in Table 2.

**Table 2.** Comparison of seven key characters of five species of *Paramesosciophilodes*.

	***Paramesosciophilodes ningchengensis***	***Paramesosciophilodes eximia***	***Paramesosciophilodes aequus***	***Paramesosciophilodes bellus* sp. n.**	***Paramesosciophilodes rarissima* sp. n.**
Length ratio of cell r and the wing	0.167 (left wing) 0.180 (right wing)	0.183 (left wing) 0.172 (right wing)	0.22 as described. But, the missing wing base was not included in wing length measurement.	0.165	0.184
Length of Sc_1_	46–47 % of the wing length	46% of the wing length	24% of the wing length as described. But, the missing wing base was not included in wing length measurement.	46–47% of the wing length	43% of the wing length
Sc_1_ ending at C	at the midlength of cell r	distad of midlength of cell r	proximad of midlength of cell r	proximad of the midlength of cell r	near the midlength of cell r
bRs vs r-m	0.6–0.7 times of length of r-m	0.5 times of the length of r-m	0.9 times of the length r-m	0.7 times of the length of r-m	0.8 times of the length of r-m
R_4+5_	slightly curved	slightly curved	strongly curved	strongly curved	strongly curved
The position of base of M_1+2_ forking vs the forking of Rs	M_1+2_ forking distinctly basad of forking of Rs	M_l+2_ forking almost at level of forking of Rs	M_1+2_ forking basad of the forking of Rs	M_1+2_ forking basad of forking of Rs,	M_1+2_ forking basad of forking of Rs
The position of base of M_1+2_ forking vs the level of Sc_1_ ending	M_1+2_ forking basad or distad of the level of Sc_1_ ending	M_1+2_ forking at the level of Sc_l_ ending	M_1+2_ forking distad of the level of the Sc_1_ ending	M_1+2_ forking slightly distad of the level of Sc_1_ ending	M_1+2_ forking slightly distad of the level of Sc_1_ ending
CuA shape	CuA strongly arched	CuA smoothly arched	CuA smoothly arched	CuA strongly arched	CuA smoothly arched
CuA ending at the posterior margin vs. Rs forking to R_2+3_ and R_4+5_	CuA ending slightly basad of Rs forking to R_2+3_ and R_4+5_	CuA ending slightly distad of Rs forking to R_2+3_ and R_4+5_	CuA ending slightly distad of Rs forking to R_2+3_ and R_4+5_	CuA ending markedly basad of Rs forking to R_2+3_ and R_4+5_	CuA ending slightly distad of Rs forking to R_2+3_ and R_4+5_

#### Paramesosciophilodes rarissima

Taxon classificationAnimaliaDipteraMesosciophilidae

Gao, Shi, Shih & Ren sp. n.

http://zoobank.org/2DC54917-79F7-4919-8B03-3BDA9BAF3B00

Figs 3 , 4

##### Etymology.

The specific name is from the Latin word of *rarissimus*, meaning rare.

##### Material.

Holotype No. CNU-DIP-NN2013145 p/c, part and counterpart. A well-preserved insect with complete body with two wings, without head and halters, in dorsoventral aspect.

##### Locality and horizon.

Daohugou Village, Shantou Township, Ningcheng County, Inner Mongolia, China, Jiulongshan Formation, late Middle Jurassic.

##### Diagnosis.

Sc_1_ ending near the midlength of cell r; bRs 0.8 times the r-m; R_4+5 _strongly curved; M_1+2_ forking basad of R_2+3_ level and distad of level of Sc_1_ ending at C; CuA strongly arched, reaching the posterior margin of the wing at the level of intersection of Rs forking to R_2+3_ and R_4+5_.

##### Description of holotype.

**Figure 3.**
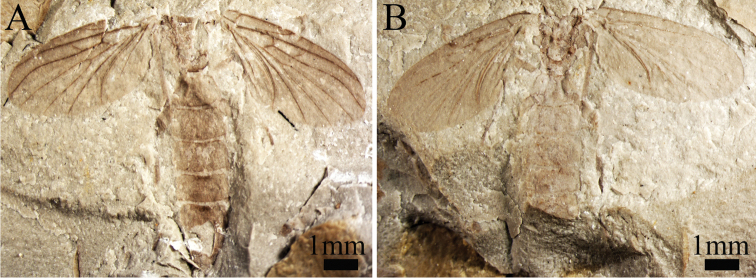
*Paramesosciophilodes rarissima* sp. n., holotype, Photographs of habitus (dorsoventral aspect): **A** No. CNU-DIP-NN2013145 p **B** No. CNU-DIP-NN2013145 c.

**Figure 4.**
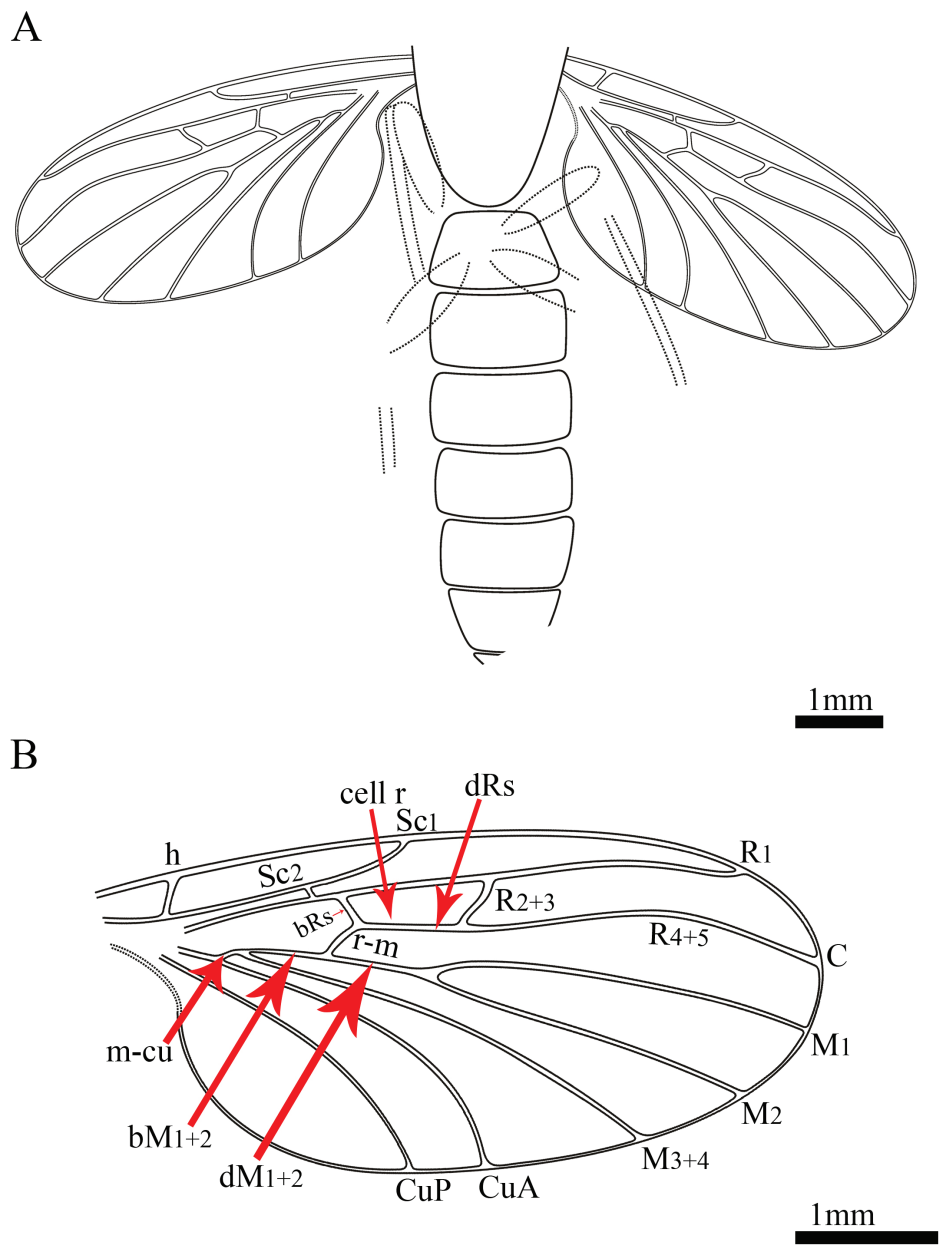
*Paramesosciophilodes rarissima* sp. n., Line drawings of holotype: **A** part **B** wing venation.

Medium-sized mesosciophilid gnats, in dorsal aspect (Figs 3 and 4A). Body length (without head and part of thorax) 7.2 mm as preserved. Legs covered with long, dense pubescence. Head, antennae, and halters not preserved. Thorax length 1.8 mm, width 1.5 mm. Mesonotum convex. Scutellum sharp, clearly projecting. Wings membranous, oblong, length 5.0 mm, width 2.2 mm, darker in color in costal area, length 2.3 times width, and not reaching the apex of abdomen at rest. Abdomen thin, subcylindrical, length 5.4 mm, width 1.5 mm, with first five segments gradually widened distally, other segments gradually narrowed terminally. Legs poorly preserved, femora thicker in the middle, covered with numerous setae.

C strong, ending beyond wing apex, at which R_4+5_ ending (Fig. 4). Sc converging with C before the level of R_4+5_. Sc_1_ elongate, slightly shorter than one-half of wing length (0.43–0.47 times the wing length), and ending far distad of the intersection of bRs and r-m. Vein h distinct and oblique. Sc_2_ developed well, starting in front of Rs, situated distinctly basal to Rs origin, arising beyond midway between h to Sc_1_ ending. Cell r relatively large (0.92 mm), approx. 0.18 times the wing length (5.0 mm). The section of R from Sc_2_ to Rs origin approx. 0.7 times the section bRs. R forking to R_1 _and Rs, then Rs to R_2+3_ and R_4+5_. Both R_1_ and R_4+5_ somewhat divergent terminally; R_2+3_ and R_4+5_ arched. Rs usually strong, arising from basal one-half of length of wing, forking to R_2+3_ and R_4+5_ beyond the forking of M_1+2_. Section bRs 0.8 times the r-m. R_1_ slightly curved, relatively long, nearly 0.5 times the wing. Both R_1_ and R_4+5_ divergent terminally. R_2+3_ curved, beyond the level of M_1_ and M_2_ forking. R_4+5_ strongly arched near its midlength. Stem of M, basad to crossvein m-cu completely reduced, with only a short segment distal to m-cu. Stem of M forking into M_1+2_ and M_3+4_. M_1+2_ forking into M_1_ and M_2_ basad of R_2+3_ level and distad to level of Sc_1_ ending at C. M_1_ arched cephalad. M_2_ nearly straight. Crossvein r-m short, curved, slightly oblique, shorter than bRs, r-m intersecting M_1+2_ and dividing M_1+2_ into bM_1+2_ and dM_1+2_. Section bM_1+2_ approx. 4.3 times the crossvein m-cu. Section dM_1+2_ approx. 1.2 times the section bM_1+2_, and longer than r-m. CuA running parallel and close to M_3+4_ basally. CuP short, slightly curved midway, reaching the posterior margin of wing at the same level as Sc_1_ ending at C.

##### Remarks.

*Paramesosciophilodes rarissima* sp. n. is distinguished from all other species of *Paramesosciophilodes* based on a combination of characters listed in Table 2.

## Discussion

As shown in Table 1, a total of 7 genera and 21 species of mesosciophilids have been reported from various localities in the Jurassic of Siberia and Kazakhstan, Lower Cretaceous of Transbaikalia, and Middle Jurassic of Inner Mongolia. One genus with 2 species was described in the Middle Jurassic Itat Formation, Siberia; 6 genera with 13 species were reported from the Middle Jurassic Jiulongshan Formation of Daohugou, Inner Mongolia, China; 2 genera with 3 species were described from the Late Jurassic Karabastau Formation in Kazakhstan; and one genus with 3 species was documented from the Early Cretaceous Zaza Formation at Baissa, Transbaikalia.

The data show that the known earliest mesosciophilids have been reported from the Middle Jurassic, while the latest ones are described from the Early Cretaceous. It seems that mesosciophilids became less diverse in the Early Cretaceous, and were possibly replaced by Mycetophilidae (Blagoderov 1993), which is supported by Zhang’s data, who listed all the mesosciophilids and mycetophilids from Daohugou, and compared them with other faunas (Zhang 2002).

## Supplementary Material

XML Treatment for Paramesosciophilodes

XML Treatment for Paramesosciophilodes bellus

XML Treatment for Paramesosciophilodes rarissima
